# A C126R *de novo* Mutation in *CYBB* Leads to X-linked Chronic Granulomatous Disease With Recurrent Pneumonia and BCGitis

**DOI:** 10.3389/fped.2018.00248

**Published:** 2018-09-11

**Authors:** Jose Antonio Tavares de Albuquerque, Edgar Borges de Oliveira Junior, Nuria Bengala Zurro, Paola Vendramini, Edson Kiyotaka Ishizuka, Daniela de Souza Paiva Borgli, Monica Soares de Souza, Antonio Condino-Neto

**Affiliations:** ^1^Immunogenic Assessoria e Diagnóstico em Saúde LTDA, São Paulo, Brazil; ^2^PENSI Institute, José Luiz Egídio Setúbal Foundation, Sabará Hospital, São Paulo, Brazil; ^3^Department of Immunology, Institute of Biomedical Sciences, University of São Paulo, São Paulo, Brazil; ^4^Hospital Federal dos Servidores do Estado, Rio de Janeiro, Brazil

**Keywords:** chronic granulomatous disease (CGD), NADPH oxidase, CYBB gene, novel mutation, pneumonia

## Abstract

Chronic granulomatous disease (CGD) is an innate immune deficiency of phagocytic cells caused by mutations that affect components of the NADPH oxidase system, with resulting impairment in reactive oxygen species production. Patients with CGD are susceptible to recurrent infections and hyperinflammatory responses. Mutations in *CYBB* lead to the X-linked form of CGD and are responsible for ~ 70% of cases. In this study, we report the case of a 2.5-year-old male patient with recurrent pneumonia and Bacillus Calmette-Guérin infection (BCGitis). As his first clinical manifestation, he presented with bullous impetigo at 18 days of age, which was followed by recurrent pneumonia and regional BCGitis. Genetic analysis revealed a *de novo* mutation in exon 5 of the *CYBB* gene: a single-nucleotide substitution, c.376T > C, leading to a C126R change.

## Introduction

The production of reactive oxygen species (ROS) is fundamental for the ability of phagocytes to eliminate microorganisms. One of the key producers of ROS in these cells is NADPH oxidase, which is composed of membrane and cytoplasmic subunits and generates superoxide by transferring electrons from NADPH to oxygen inside phagolysosome (1–3). Of these components, flavocytochrome *b*_558_, which is located at the membrane, consists of gp91phox and p22phox; p47phox, p67phox, and p40phox are located in the cytoplasm (4). The genes encoding gp91phox, p22phox, p47phox, p67phox, and p40phox are *CYBB, CYBA, NCF1, NCF2*, and *NCF4*, respectively. When activated, the cytoplasmic subunits translocate to the membrane, where the NADPH oxidase complex catalyzes the conversion of molecular oxygen (O_2_) to superoxide anion (${\text{O}}_{2}^{-}$) and other reactive oxygen intermediates (5).

Chronic granulomatous disease (CGD) is an inherited immunodeficiency disease caused by defects in NADPH oxidase. Mutations in all five structural genes of the NADPH oxidase complex have been implicated in CGD, affecting 1 in 250,000 live births in the USA. Mutations in *CYBB*, which cause X-linked CGD (XL-CGD), are responsible for ~ 70% of CGD cases in Latin America (2). The hallmark of this immunodeficiency is the development of chronic inflammatory granulomas, hyperinflammatory responses, and recurrent infections (1, 3, 4). Indeed, lung infections are prevalent in CGD patients and may be complicated by granulomatous inflammation, persistent hilar or mediastinal lymphadenopathy, and pulmonary fibrosis (1, 2, 6).

Common pathogenic agents in these cases include *Staphylococcus aureus, Escherichia coli, Aspergillus spp, Candida spp, Klebsiella spp*, and *Burkholderia cepacia*. Other symptomatic occurrences involve colitis/enteritis or granulomatous obstruction of either the gastric outlet or urinary tract (1, 2, 6). Approximately 30% of patients in Latin America have adverse reactions to Bacillus Calmette-Guérin (BCG) vaccinations (2).

In this study, we report a male patient with X-linked CGD with recurrent pneumonia, regional BCG vaccine dissemination and a *de novo* mutation in *CYBB* that results in the single-amino acid substitution C_126_R in the gp91phox protein.

## Case report

The proband (2.5 years old) was a male child from a non-consanguineous family with no immunodeficiency history. The mother reported two abortions prior to the birth of the child by cesarean section at 37 weeks. The patient was discharged from the hospital after 48 h without any clinical manifestations. The first clinical manifestation was bullous impetigo at 18 days of age, which was treated with oxacillin and amikacin for 28 days.

**Figure 1:**
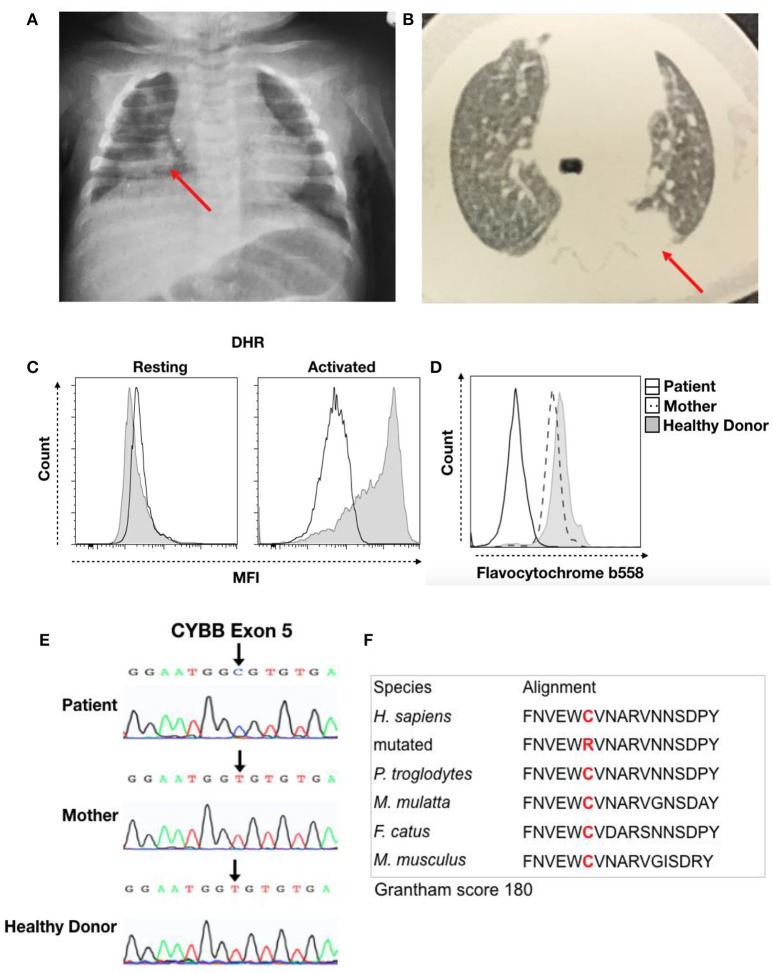
Clinical record of the patient with CGD. **(A)** Chest X-ray from the first hospitalization at 3 months of age and **(B)** thorax computed tomography at 5 months of age. The red arrow indicates the local lesion. **(C)** DHR of granulocytes in resting or activated states from the patient (Black Line) and a healthy donor (Solid Gray). **(D)** Flavocytochrome *b*_558_ expression in granulocytes from the patient (Black Line), his mother (Dash Line), and a healthy donor (Solid Gray). **(E)** Sequencing results for the *CYBB* gene. Chromatographs for the patient (Top), his mother (Middle), and a healthy donor (Bottom). The black arrow shows a single-nucleotide substitution, c.376T > C, that results in a C_126_R change. **(F)** We performed sequence analysis using MutationTaster to observe amino acid conservation in alignment with other species and obtain the Grantham matrix score.

Thereafter, the child was hospitalized with pneumonia, with chest X-ray showing a hypotransparent lesion in the lower right lobe (Figure 1A). The patient was treated with antibiotics for 35 days (5 days of clavulanate, 10 days of cefuroxime, and 20 days of cefepime and vancomycin). Due to his bullous impetigo and pneumonia, with a delayed response to treatment and low weight development, primary immunodeficiency was suspected, and the patient was discharged from the hospital with sulfamethoxazole-trimethoprim (TMP-SMX) and itraconazole prophylaxis at 3 months of age. At 5 months of age, he developed axillary lymphadenitis caused by a BCG vaccination reaction. His regional BCG infection (BCGitis) improved after isoniazid and ethambutol treatment for 45 days, with a normal BCG scar and without BCG dissemination.

The patient then developed a persistent subcutaneous nodule in his upper limb at 6 months of age. Biopsy showed granulomatous dermatitis with epithelioid histiocytes, few lymphocytes and no giant cells, and it was negative for BK and fungi. At 9 months of age, a new episode of pneumonia developed. Thorax computed tomography (CT) revealed a hypotransparent lesion in the left upper lobe that improved with cefuroxime treatment. However, chest X-ray confirmed a consolidation lesion in the left upper lobes (Figure 1B), and subcutaneous nodular lesion biopsy showed nonspecific granular cells. The pulmonary lesions improved with treatment. His prophylaxis treatment was suspended at 10 months of age.

The patient developed bacterial cervical adenitis, which was treated with amoxicillin and clavulanate for 21 days, with total resolution when he was 1 year old. However, 1 month later, he started to present intermittent fever and received a new diagnosis of pneumonia and large consolidation lesions. The pediatric service indicated an oncology study, which revealed a normal myelogram. There was no evidence of neoplasm. Pulmonary biopsy showed a granuloma lesion with areas of central necrosis that was not caseous and was negative for BK and fungi; abdominal ultrasound was normal, and PCR was negative for BK in gastric lavage and culture. Immunological tests revealed hypergammaglobulinemia and a subpopulation of CD4, CD8, and CD19 lymphocytes above the 90th percentile. According to chest X-ray, the parents had no contact with BK. His lesion improved markedly after prolonged antibiotic therapy, with the presence of a hypotransparent lesion.

The hypothesis for primary immunodeficiency in phagocytes was investigated using the dihydrorhodamine (DHR) assay, and the abnormal result of ROS production in granulocytes after stimulation was suggestive of CGD (Figure 1C). We then performed flavocytochrome *b*_558_ staining to observe gp91phox and p22phox expression at the membrane by flow cytometry. The lack of membrane flavocytochrome *b*_558_ in this patient in comparison to his mother and a healthy donor suggested gp91phox and p22phox expression failure (Figure 1D). In addition, we sequenced the *CYBB* gene to confirm CGD diagnosis. The sequence data were analyzed using NCBI (*www.ncbi.nlm.nih.gov*) and Ensembl SNP (*https://www.ensembl.org/index.html*) databases. This analysis confirmed a missense mutation (c.376T > C) in exon 5 that leads to a C126R substitution in the gp91phox protein; conversely, the mother and healthy donor presented the normal sequence, confirming a diagnosis of CGD caused by a *de novo* mutation (Figure 1E). Cysteine 126 is conserved, and this C126R substitution has a 180 score according to the Grantham matrix in MutationTaster (http://mutationtaster.org) (Figure 1F). At follow-up visits, the patient showed improvement with long-term sulfamethoxazole and itraconazole treatment for CGD prophylaxis.

## Discussion

CGD is a rare, primary genetic immunodeficiency resulting from a defect in phagocytic cells that leads to increased susceptibility to bacterial and fungal infections, such as abscess, pneumonia, skin ulcers, sepsis, and osteomyelitis (1–3, 7). A common clinical manifestation in patients with CGD is several infections by catalase-producing microorganisms (2, 4). The most commonly reported invasive infectious agents are *Staphylococcus, Serratia ssp*, and *Aspergillus ssp*. *S. aureus* is the major cause of infection and subcutaneous/liver abscess formation in these patients (1, 8).

Skin manifestations in CGD patients are reported with systemic or deep infections that affect periorbital and perioral skin as well as the nares, scalp, neck, and retroauricular fold. In most cases, atopic dermatitis is prevalent, whereas bullous impetigo is rarely reported (9, 10).

Lung infections are frequent, occurring in more than 80% of CGD patients (2, 11). The high incidence of pneumonia in CGD is caused by many bacteria and fungi and may be complicated by abscess formation or pleural empyema in up to 20% of patients (6, 7, 11, 12). Thus, respiratory complications are a significant cause of mortality and morbidity (4). Our patient had been developing recurrent pneumonia since he was 3 months old, and it was the main cause of his hospitalization. Although no bacteria or fungi were isolated from this patient, antibiotic treatment and TMP-SMX and itraconazole prophylactic treatment helped the patient recover in all episodes and decreased his number of infections.

Prophylaxis with TMP-SMX for bacterial infections and with itraconazole for fungal infections has been routinely used since the 1970s (13). Prophylaxis treatment decreases the incidence of non-fungal infections in patients with autosomal and X-linked CGD without increasing the incidence of fungal infections. More than 40% of CGD cases remain infection free for more than 1 year with TMP-SMX prophylaxis (14).

The median age at onset of symptoms in CGD patients, with episodes of infection or noninfectious complications, is 4 months of age. However, tuberculosis-endemic countries provide BCG vaccination within the first month of life, and patients with immunodeficiency may quickly develop adverse reactions to BCG vaccination (6). Indeed, studies have shown that ~ 30% of CGD patients suffer from BCG reactions after vaccination (2). Furthermore, patients with any form of CGD can fail to develop a protective immune response against *Mycobacterium* species and may develop tuberculosis later in life. Clinically, BCG infection has in some cases been the first manifestation of CGD in Brazil. Nevertheless, our patient developed only regional BCGitis at 5 months of age, which was successfully treated with antibiotics, and BCG was not disseminated after the episodes of bullous impetigo and pneumonia.

Mutations in all five genes encoding components of the NADPH enzyme complex have been reported. The most affected gene is *CYBB*; mutations in this gene lead to an absence of or nonfunctional gp91phox, which is associated with a lack of ROS production by NADPH oxidase in phagocytes (2, 5). An international XL-CGD database includes over 680 mutations in the *CYBB* gene (15). In the present study, we reported a *de novo* mutation leading to X-linked CGD in a male patient. The mutation in exon 5 of *CYBB* leads to a C126R missense mutation in gp91phox that was not found in the patient's mother but has been reported in other CGD patients (16). This cysteine residue is responsible for forming disulfide bonds, which are important for protein folding. Therefore, this missense mutation may abolish protein expression, as observed for the flavocytochrome *b*_558_ histogram, and may result in abnormal DHR results. Additionally, patients with mutations in *CYBB* have been reported to be predisposed to tuberculous mycobacterial disease, corroborating our case report (17).

In summary, we report a *de novo* mutation, C126R, in gp91phox that led to X-linked CGD in a patient with recurrent pneumonia and regional BCGitis who had no family history, suggesting primary immunodeficiency. Clinicians should consider the possibility of a *de novo* mutation under such circumstances, and the results of this study highlight the relevance of genetic diagnosis for definitive genetic counseling for families with children who do not have any indications regarding inherited disease.

## Ethics statement

This study was approved by the Ethics Committee for Research in Humans at the Institute of Biomedical Sciences of the University of Sao Paulo in accordance with the Declaration of Helsinki, with written informed consent from the mother of the pediatric patient. The patient, his mother, and the healthy donor provided written informed consent before the study began. Written and informed consent for publication of this case report was obtained from the mother of the patient.

## Author contributions

JdA, EdOJ, and NZ designed and conducted the experiments and wrote the manuscript. EI and PV conducted the experiments. DB and MdS performed clinical and laboratory data collection and analysis. DB, MdS, and AC-N designed the experiments and wrote and reviewed the manuscript.

### Conflict of interest statement

The authors declare that the research was conducted in the absence of any commercial or financial relationships that could be construed as a potential conflict of interest.
